# A Systematic Review of Clinical Outcomes and Technical Considerations: Endoscopic Spine Surgery for Primary Spinal Tumors

**DOI:** 10.3390/jcm15124623

**Published:** 2026-06-14

**Authors:** MaryLourdes Andreu, Anshul Ratnaparkhi, Long Di, Robert Kamil, Khushi H. Shah, Tyler M. Cardinal, Seth S. Tigchelaar, Adham M. Khalafallah, Gregory W. Basil

**Affiliations:** 1 Miami Project to Cure Paralysis, University of Miami Miller School of Medicine, Miami, FL 33136, USA; mxa1485@med.miami.edu; 2 Medical Scientist Training Program, University of Miami Miller School of Medicine, Miami, FL 33136, USA; 3 Department of Neurosurgery, University of Miami Miller School of Medicine, Miami, FL 33136, USA; aratnaparkhi@med.miami.edu (A.R.); lxd545@med.miami.edu (L.D.); rxk512@miami.edu (R.K.); khs55@med.miami.edu (K.H.S.); tmc204@miami.edu (T.M.C.); sst98@miami.edu (S.S.T.); gbasil@med.miami.edu (G.W.B.)

**Keywords:** endoscopic spine surgery, primary spinal tumors, minimally invasive, intradural tumors, uniportal endoscopy, biportal endoscopy, full-endoscopic surgery

## Abstract

**Background:** Endoscopic spine surgery (ESS) is an established minimally invasive approach for degenerative spinal conditions. Advances in instrumentation and visualization have expanded its application to spinal tumor resection. This review synthesizes reported clinical outcomes and technical considerations of ESS for primary spinal tumors. **Methods:** PubMed was queried from 2000 to 2025 for studies reporting endoscopic resection of primary spinal tumors. Studies involving metastatic disease or non-resective interventions were excluded. Data were descriptively analyzed given heterogeneity and limited sample size. **Results:** Eleven patients across seven studies were included (mean age = 50.3 years). Pathologies comprised schwannoma (n = 5), meningioma (n = 3), osteoid osteoma (n = 2), and Ewing sarcoma (n = 1). Seven tumors were intradural extramedullary (63.6%) and four were extradural (36.4%); no intramedullary lesions were included. Of the seven intradural cases, one was performed via uniportal full-endoscopic technique, one via biportal endoscopy, and five via tubular retractor-assisted endoscopy. Across all eleven patients, gross total resection was achieved in 90.9% of cases. Gross total resection was achieved in 100% of cases in which it was the operative intent (10/10); the remaining case was a planned biopsy of recurrent Ewing sarcoma. One transient postoperative lower extremity weakness was reported; no cerebrospinal fluid leaks, reoperations, or perioperative deaths occurred. No recurrences were observed across a mean follow-up of 21.9 months (range 4–48 months), though this duration may be insufficient to assess long-term recurrence for slow-growing tumors such as meningioma and schwannoma. **Conclusions:** ESS of primary spinal tumors appears feasible and safe in carefully selected cases, particularly for small, well-circumscribed lesions in favorable anatomical locations. Intradural resection introduced distinct technical challenges, including irrigation management and dural closure, which influence platform selection. These findings are limited by small sample size, short follow-up, and likely publication bias. ESS should be considered an emerging minimally invasive option rather than a replacement for established microsurgical approaches. Prospective comparative studies are needed to better define its role in spinal oncology.

## 1. Introduction

Primary spinal tumors comprise a heterogeneous group of neoplasms arising within the intramedullary, intradural extramedullary, and extradural compartments of the spine [1,2,3]. Although less common than metastatic disease, these lesions can result in progressive neurological deficits, spinal instability, chronic pain, vertebral collapse, and cord compression [4,5]. Management is inherently multimodal and depends on tumor histology, anatomic location, and oncological stage, ranging from intralesional resection for benign, well-circumscribed lesions to en bloc resection and adjuvant therapies for primary malignant tumors [6,7,8].

Surgical resection remains a cornerstone of management for many primary spinal tumors; however, the morbidity associated with traditional open approaches is well established [2,9,10,11]. For the benign intradural extramedullary and focal extradural lesions that constitute the majority of surgically treated primary tumors, resection through open laminectomy with paraspinal muscle stripping and partial facetectomy remains the standard of care [2,12,13,14,15]. While effective, these techniques are associated with postoperative pain, prolonged recovery, wound-related complications, and iatrogenic disruption of spinal stability, sometimes necessitating instrumented fusion. In patients with limited physiological reserve or those requiring timely adjuvant therapy, this operative burden may adversely affect overall recovery and treatment sequencing. Conversely, other minimally invasive options, including percutaneous vertebroplasty with interventional tumor removal for metastatic vertebral disease [16] and percutaneous radiofrequency ablation or core excision for select extradural lesions such as osteoid osteoma [17], address pain palliation or focal lesion destruction rather than the broader operative goals of primary spinal tumor surgery: resection [18].

Endoscopic spine surgery (ESS) has emerged as a minimally invasive alternative that minimizes soft tissue disruption and preserves key stabilizing structures, including the posterior tension band, facet joints, and paraspinal musculature. By operating through narrow working corridors with enhanced visualization, minimized osseous and tissue manipulation, ESS preserves the facets, posterior tension band, and paraspinal musculature that open and microsurgical approaches routinely sacrifice for adequate exposure [19,20,21]. Although ESS is associated with higher operative costs compared to open surgery, emerging evidence in degenerative spine surgery suggests that this may be balanced with lower overall societal costs, owing to shorter hospital stays and faster return to work [22,23]. These advantages have prompted growing interest in extending ESS to spinal oncology.

However, the application of ESS to primary spinal tumors presents distinct challenges that are not encountered in degenerative pathology. Unlike metastatic disease, where decompression is often palliative, resection of primary tumors frequently aims for gross total resection and may require intradural access, delicate neural dissection, and dural closure. These technical demands introduce considerations unique to oncologic surgery, including irrigation management after durotomy, maintenance of visualization in confined spaces, and limitations of endoscopic instrumentation.

Despite increasing reports of endoscopic tumor resection, the current literature remains limited and fragmented. Prior reviews have combined primary and metastatic spinal tumors into a single cohort [24], obscuring differences in surgical goals, technical complexity, and outcome expectations. As a result, the role of ESS in the management of primary spinal tumors remains poorly defined.

Accordingly, the present study aims to synthesize the available literature on endoscopic resection for patients with primary spinal tumors. Specifically, we aim to examine the impact of ESS on clinical outcomes, technical feasibility, and platform-specific considerations, compared with traditional open or minimally invasive surgical approaches when available in the literature. By isolating this distinct population, this review seeks to clarify the current role of ESS in the management of primary spinal tumors and to identify the technical adaptations required for its safe application.

## 2. Materials and Methods

### 2.1. Study Design and Protocol Reporting

This study was conducted as a systematic review following the PRISMA (Preferred Reporting Items for Systematic Reviews and Meta-Analyses) 2020 guidelines for systematic reviews and meta-analyses (Supplementary Materials). Given the exploratory nature of the topic and limited available data, a protocol was not prospectively registered. No meta-analysis was performed.

### 2.2. Search Strategy

A systematic search of PubMed was performed to identify studies published between January 2000 and December 2025. The search combined MeSH terms and free-text keywords related to spinal tumors, endoscopy, and spine surgery. The full search string was: (“Spinal Neoplasms”[MeSH] OR “spinal tumor”[tiab] OR “spinal tumour”[tiab] OR “spine tumor”[tiab] OR “spine tumour”[tiab] OR “intradural tumor”[tiab] OR “extradural tumor”[tiab] OR “epidural tumor”[tiab] OR “metastatic spinal tumor”[tiab] OR “primary spinal tumor”[tiab]).

AND (“Endoscopy”[MeSH] OR “endoscopic surgery”[tiab] OR “endoscopic resection”[tiab] OR “endoscopic approach”[tiab] OR “minimally invasive endoscopic”[tiab] OR “full endoscopic”[tiab] OR “biportal endoscopic”[tiab]) AND (“Spine”[MeSH] OR “spinal”[tiab] OR “vertebral”[tiab]).

Reference lists of included studies were manually screened to identify additional eligible articles.

### 2.3. Eligibility Criteria

Studies were included if they met the following criteria:

1. Patients with primary spinal tumors (extradural or intradural) tumors
2. Patients undergoing endoscopic spine surgery (full-endoscopic [FESS] or endoscopic-assisted [EASS])
3. Reporting ≥ 1 prespecified outcome of interest: length of stay (LOS), postoperative complications, reoperation, time to adjuvant therapy, or survival
4. Full-text available in English
5. Eligible study designs: randomized or non-randomized cohorts, case series, or case reports

Exclusion criteria:

1. Studies focused exclusively on metastatic spinal tumors
2. Interventions without surgical resection (e.g., radiotherapy or chemotherapy only)
3. Editorials, letters, technical reports, conference abstracts, systematic reviews cadaveric/anatomic studies
4. Out-of-scope procedures (thoracoscopic approaches including video-assisted thoracoscopic surgery [VATS], laparoscopic, or robotic-only surgery)
5. Non-English language publications

### 2.4. Study Selection & Data Extraction

Two reviewers (MA, LD) independently screened titles, abstracts, and full texts for eligibility. Discrepancies were resolved through consensus. Extracted variables included: study design, country of origin, sample size, patient demographics, tumor level and pathology, dural compartment, type of endoscopic technique (FESS vs. EASS), extent of resection, LOS, complications, reoperation, time to adjuvant therapy, follow-up duration, and survival.

### 2.5. Quality Appraisal

Methodological quality was evaluated using the Joanna Briggs Institute (JBI) critical appraisal tools for cases series (10 items checklist) and case reports using the JBI Case Report (8 items checklist) [25,26]. Given the descriptive nature of the included studies, quality appraisal was used to contextualize findings rather than to exclude studies.

### 2.6. Outcomes

Given the small sample size, heterogeneity of study designs, and predominance of case reports, a meta-analysis was not performed. Outcomes were summarized descriptively using counts, proportions, and ranges where appropriate. These included the total number of patients in each study, sex female: male ratio, number of criteria met in the JBI tool (case series out of 10, case report out of 8), frequency of cervical, thoracic, lumbar, sacral primary tumors (%), pathology of primary tumor (%), type of surgery (full endoscopic, endoscopic assisted), tumor resection extent, anatomical position and dural location of tumor, length of hospital stay (days), post-operative complication rate per study (%), reoperative rate (%), time to adjuvant therapy (days).

## 3. Results

### 3.1. Study Selection and Characteristics

**Figure 1:**
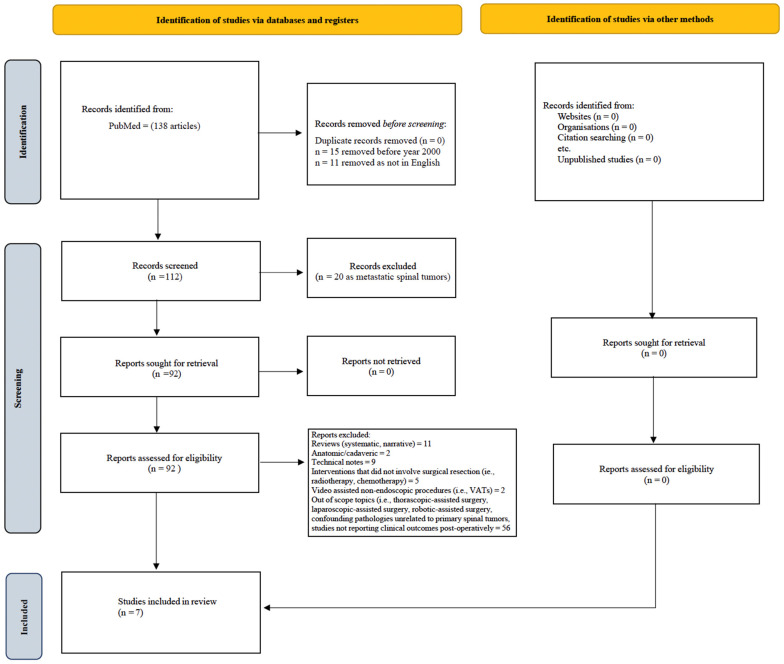
PRISMA flow diagram illustrating study selection process.

The PubMed search initially yielded 138 articles. After excluding non-English publications and those published before 2000, 112 articles remained. Following full-text screening, 7 studies met the inclusion criteria for this systematic review [27,28,29,30,31,32,33,34] (Figure 1).

The included studies consisted of 1 case series (14.3%) and 6 case reports (85.7%). Methodological quality assessment using the JBI tools confirmed low risk of bias: the case series satisfied 9/10 criteria, while all case reports satisfied 8/8 criteria. The case series received a score of 9/10 as the authors did not directly explain why statistical analyses tests were not performed. Studies originated from diverse regions: China (n = 2, 28.6%), South Korea (n = 1, 14.3%), Germany (n = 1, 14.3%), Puerto Rico (n = 1, 14.3%), Russia (n = 1, 14.3%), and the United States (n = 1, 14.3%). A total of 11 patients were included across the 7 studies. The mean age was 50.3 years, and the sex distribution was 6 females (54.5%) and 5 males (45.5%).

### 3.2. Clinical Presentations

Presenting symptoms varied according to tumor location, and included reported lumbar pain with lower-limb numbness (n = 1), thoracic pain with numbness and weakness (n = 2), and lumbar pain alone (n = 2) [27]. Case reports similarly described heterogenous presentations including: clumsiness, hand tingling, gait disturbance, bilateral lower-extremity weakness [28] acute nocturnal lumbar pain relieved by NSAIDs [29] long-standing lumbar pain, bilateral radiculopathy, lower-limb weakness, hyperesthesia [30] thoracic pain and local tightness, nocturnal exacerbation relieved by NSAIDs [31] constant leg pain with moderate foot extensor paresis and positive Lasègue’s sign [32] mid-thoracic back pain with posterior rib pain and subjective lower-limb numbness [33] (Table 1).

### 3.3. Tumor Characteristics

The most common tumor levels were lumbar (n = 6, 54.5%), followed by thoracic (n = 4, 36.4%) and cervical (n = 1, 9.1%). Histopathologies included schwannoma (n = 5, 45.5%), meningioma (n = 3, 27.3%), osteoid osteoma (n = 2, 18.2%), and sarcoma (n = 1, 9.1%). Tumor positions were variable, including dorsal (27.3%), ventrolateral (27.3%), lateral (18.2%), ventral (9.1%), dorsolateral (9.1%), and foraminal (9.1%). Dural locations were intradural (n = 7, 63.6%) and extradural (n = 4, 36.4%) (Table 2).

### 3.4. Surgical Approach and Extent of Resection

Procedures included FESS (n = 5, 45.5%) and EASS (n = 6, 54.5%). Gross-total resection was achieved in 10 patients (90.9%), while 1 patient (9.1%) underwent biopsy only without intent for complete resection. No subtotal resections were reported. Specified irrigation pressure management was reported in two of the seven articles as 30 mmHg and the minimum necessary to prevent venous bleeding [28,30]. Dural closure techniques were reported in two of the seven articles, including nylon 7-0 and separated stitches reinforced with overlay dural patch [28,30]. Surgeon experience level was not reported in any of the seven articles.

### 3.5. Clinical Outcomes

The reported LOS ranged from less than one day to seven days. One patient developed temporary lower-limb weakness attributed to tight tumor–dural adhesion from a meningioma, which resolved at follow-up [27]. The remaining patients did not experience postoperative complications. No reoperations were required across the series. No tract recurrence or corridor implantation was reported across the included cases; however, ten of eleven tumors were benign and the single malignant lesion (Ewing sarcoma) underwent biopsy rather than attempted resection. All eleven patients survived the index surgery and remained alive at last follow-up, which ranged from 4 to 48 months. Importantly, no cases of tumor recurrence or progression were observed during the follow-up period (Table 1).

## 4. Discussion

This systematic review synthesizes the available literature on endoscopic resection of primary spinal tumors and demonstrates that ESS appears feasible and safe in carefully selected patients. Across 11 reported cases, gross total resection was achieved in the majority of patients, with minimal complications and no reported recurrences within the available follow-up. However, these findings must be interpreted within the context of highly selected tumor characteristics and limited evidence, rather than as evidence of broad applicability.

### 4.1. Case Selection and Current Clinical Role

A consistent pattern across the included studies was deliberate case selection. Excluding one planned biopsy of recurrent Ewing sarcoma, gross total resection was achieved in all 100% of cases (10/10). No recurrences were observed within the available follow-up. Complications were rare and transient, occurring in only one patient, who experienced mild lower limb weakness related to tight adhesion between a meningioma and the dura, which resolved on subsequent follow-up [27]. No cerebrospinal fluid leaks, wound infections, or reoperations were reported in any case. The favorable outcomes reported across this review must be interpreted in the context of strict patient selection. The tumors resected endoscopically were predominantly small, well-circumscribed, and benign, representing the most favorable end of the primary spinal tumor spectrum, reflecting appropriate matching of technique to indication rather than broad applicability. The present findings demonstrate technical feasibility and identify potential advantages in structural preservation and close-range visualization, but the limited sample size and absence of comparative arms do not provide sufficient evidence to establish equivalence with open or microsurgical approaches. Endoscopic resection should be understood as an emerging option within the minimally invasive alternative for carefully selected primary spinal tumors rather than a replacement for established techniques.

### 4.2. Extradural Tumors: Structural Preservation and Stability

Four of the eleven cases involved extradural pathology, including two vertebral body osteoid osteomas [29,31], one foraminal neurinoma resected through a transforaminal approach without durotomy [32], and one recurrent ventral epidural Ewing sarcoma in a previously irradiated field [33]. No complications, reoperations, or recurrences were reported across the cases. 

The principal advantage of endoscopic resection lies in preservation of spinal stability [35,36]. Endoscopic approaches, by virtue of operating through narrow corridors with targeted bony removal, retain the structural elements that maintain spinal stability [37]. This osseous preservation often precludes the need for fusion instrumentation augmentation that is often required to manage structural defects created by more extensive resection [38]. This advantage was particularly apparent in patients with extradural bony tumors, in which endoscopic corridors facilitated complete resection without compromising stability and mitigated the need for concomitant arthrodesis required by open techniques [29,31]. Both endoscopic osteoid osteoma cases achieved complete resection through corridors that preserved stability without supplemental fusion, owing to minimal osseous and tissue trauma due to the narrow endoscopic corridor [39]. Conventional open resection of vertebral body osteoid osteomas frequently requires extensive posterior element removal or corpectomy, with one series reporting that 81.3% of patients required instrumented fixation after open surgical resection [31]. At one-year follow-up, both patients demonstrated complete pain resolution, radiographic evidence of bone remodeling without recurrence, and no spinal instability [29,31]. These findings highlight the potential role of ESS in motion-preserving tumor surgery, particularly for focal lesions in the posterior elements or vertebral body accessible through established endoscopic corridors.

### 4.3. Intradural Tumors: Technical Constraints and Adaptations

Seven of the eleven included cases involved intradural resection. Endoscopic approaches achieved gross total resection in all seven cases through limited posterior bony removal [27,28,30], with no reported postoperative instability or kyphosis during available follow-up. However, intradural resection introduced operative constraints not encountered in the extradural cohort, most notably irrigation pressure management after durotomy and endoscopic dural closure.

ESS relies on continuous saline irrigation to maintain visualization and limit venous bleeding. While this is typically well tolerated in extradural degenerative procedures, durotomy allows irrigation to communicate directly with the intradural space, in which excessive pressure with irrigation risks elevation of intracranial pressure. Two of the intradural studies specifically addressed this by reducing irrigation pressure after durotomy to the minimum necessary for venous hemostasis [30] or approximately 30 mmHg [28], with additional measures including maintaining open endoscopic outflow and head-of-table elevation to mitigate intracranial pressure transmission. Although none of the included studies employed objective intraoperative ICP monitoring, this may serve as a useful adjunct in select patients, offering real-time detection of irrigation-related intracranial hypertension that surrogate measures such as outflow patency and patient positioning cannot directly provide. Hemostasis under continuous irrigation remains a separate technical constraint, as bleeding obscures the endoscopic field and the hemostatic tools available through endoscopic working channels are more limited than those in open surgery. No highly vascular lesions were included in the present study, and future work is required to establish the safety of ESS in this cohort.

Dural closure under endoscopic conditions remains an unsolved technical challenge, with no established gold standard approach. Primary suture repair, while routine in open microsurgery, is complicated by the confined working space of endoscopic corridors, particularly when the durotomy is in a lateral or otherwise difficult-to-access location. All intradural studies in the present cohort that described their closure methods achieved primary suture repair, with supplementary measures including overlay dural patches and absorbable artificial dura employed as secondary seals when concerned for risk of cerebrospinal fluid leak. Despite this technical challenge, no cerebrospinal fluid leaks were reported in any intradural case, which may be attributable to the surgeon experience. Hagel and Van Isseldyk acknowledged that tissue fragility or limited surgeon experience may preclude reliable endoscopic suturing, in which case synthetic dural substitutes, autologous tissue grafts, or conversion to microsurgery become necessary fallbacks [30]. Taken together, irrigation management and dural closure represent technical demands specific to intradural endoscopic tumor resection that are not encountered in degenerative endoscopic practice, and their successful management across all seven cases in this cohort required platform-specific adaptations and deliberate intraoperative strategy rather than straightforward application of existing endoscopic techniques.

### 4.4. Endoscopic Platforms and Operative Strategy

The choice of endoscopic platform has important implications for surgical strategy. Uniportal systems offer minimal tissue disruption but are limited by single-instrument access, restricting bimanual dissection and complicating hemostasis and dural repair.

In contrast, endoscopic-assisted and biportal approaches enable bimanual instrumentation, improving the ability to perform delicate neural dissection and dural closure. In this review, the majority of intradural cases were performed using these approaches, reflecting their technical advantages in more complex tumor resections [40,41,42,43,44].

Uniportal endoscopic spine surgery uses a single coaxial cannula 7 to 7.5 mm in diameter that houses both the endoscope and a single working channel. This platform offers the smallest tissue footprint with small skin incisions and minimal blood loss. All four extradural cases used uniportal approaches, though the surgical corridors varied. Kravtsov et al. [32] used a lumbar transforaminal approach analogous to those employed for endoscopic discectomy, while Telfeian et al. [33] adapted the transforaminal concept to the thoracic spine for biopsy and ventral decompression. The two osteoid osteoma cases required navigated posterior approaches to the vertebral body. The principal limitation of this approach is single-instrument access, which precludes simultaneous countertraction and dissection. While endoscopic spine surgeons have become accustomed to this constraint during extradural procedures, intradural tumor resection introduces the demands of neural dissection, hemostasis under irrigation, and dural closure through a single working channel, substantially increasing technical complexity. Only one intradural tumor in this cohort was resected through a uniportal platform by repurposing the working sheath bevel as a second dissector to approximate bimanual technique, though they acknowledged that tissue fragility or limited surgeon experience may render this approach infeasible.

The remaining six intradural cases used platforms that separated visualization from instrumentation, restoring bimanual capability that the uniportal corridor does not readily accommodate. Tubular retractor-assisted endoscopy introduces a hand-held endoscope through a wider fixed retractor alongside independently manipulated instruments, permitting bimanual dissection and formal dural closure within a single corridor. This builds on a well-established tubular retractor corridor for spinal tumor resection [45]. However, the substitution of endoscopic for microscopic visualization within this corridor provides superior illumination and an expanded field of view by positioning the optic directly at the surgical field rather than at distance through the retractor [46]. Five of seven intradural tumors in the cohort were resected through this approach, including the largest at 35 × 13 mm.

Biportal endoscopy builds on this concept by separating the endoscope and working instrument into fully independent portals, decoupling visualization from instrumentation entirely and allowing the surgeon to angle and advance the optic toward structures of interest without displacing the working instrument. Jung and Kim modified this approach for an anterolateral C2–C3 meningioma, adding a third portal for instrument triangulation to achieve dural opening, gross total resection, and primary dural closure with 7-0 nylon sutures at a high cervical level where preservation of posterior structural integrity carries particular biomechanical significance. While no direct comparison of platforms for watertight dural closure exists in the included literature, the case distribution suggests an implicit platform preference: six of seven intradural cases were performed via bimanual platforms (one biportal, five tubular retractor–assisted), reflecting the technical advantages of bimanual instrumentation where simultaneous countertraction and suturing are difficult through a single working channel.

Beyond endoscopic platforms, robotic-assisted systems such as the Da Vinci have been explored in spine surgery and offer improved three-dimensional visualization and instrument dexterity that may enhance precision in tumor resection [47]. However, the evidence base remains limited to small case series without comparison to endoscopic or open approaches, and robotic platforms remain largely outside current primary spinal tumor practice, representing a parallel trajectory in minimally invasive spine surgery rather than an established alternative.

### 4.5. Study Limitations of the Current Evidence

Several limitations constrain these results. All included studies are case reports or a single small case series with a total sample of 11 patients and no comparative arms. Certain tumor subtypes were not represented in these included cases, particularly vascular lesions and malignant primary spinal tumors, which influence surgical decision-making. Specifically, these tumors require wider exposure, more aggressive resection, and greater hemostatic control which may not be feasible in the endoscopic corridor. The findings of this systematic review, therefore, may not be generalizable to those tumor types, underscoring the need for future studies to assess the safety and feasibility of ESS in these complex tumors. Outcome measures were unstandardized, with pain, neurological status, and functional recovery assessed by different instruments across studies, and no validated quality-of-life measures were employed. Follow-up ranged from 4 to 48 months, inadequate to assess long-term recurrence for slow-growing tumors such as meningioma and schwannoma. Six of seven included studies identified the steep learning curve as a significant barrier to broader adoption, yet no study reported surgeon case volume or prior endoscopic experience, and the proficiency threshold required for safe endoscopic intradural tumor resection remains undefined. While recent literature has suggested that approximately 32 (uniportal and biportal) cases are required to meet proficiency, [48] the included studies did not clearly delineate the endoscopic proficiency of the primary surgeon. As such, the results reported herein cannot necessarily be extrapolated to early endoscopic practitioners. Rather, these cases likely represent the highest level of complexity of endoscopic surgery. Additionally, the search was limited to English-language publications, potentially excluding relevant literature from East Asian and European centers where endoscopic spine surgery is widely practiced. Future investigation should prioritize prospective multicenter registries with standardized oncologic staging, validated patient-reported outcomes, and mandatory reporting of surgeon endoscopic experience. Additionally, publication bias toward successful cases is likely, potentially overestimating the safety and efficacy of ESS. Given the biological diversity of these tumors, future studies should consider stratification by tumor type and compartment involvement. Future investigations should also quantify preservation of stabilizing structures through detailed operative reporting, pre- and post-operative imaging, and by monitoring delayed instability and fusion rates. Such data would provide evidence that ESS contributes to spinal stabilization compared with alternative approaches. These limitations notwithstanding, this review represents a systematic review of endoscopic resection applied exclusively to primary spinal tumors, demonstrating its feasibility in carefully selected cases and identifying the technical adaptations required to extend endoscopic surgery from its established degenerative applications into the intradural oncologic domain.

## 5. Conclusions

ESS for primary spinal tumors appears feasible and safe in carefully selected patients, with gross total resection achieved in 10 of 11 cases, no cerebrospinal fluid leaks, no reoperations, and no recurrences within the available follow-up. The technique offers potential advantages in tissue preservation and postoperative recovery, particularly for small, well-circumscribed lesions in anatomically favorable locations. Tumors with intradural components introduce distinct technical challenges, including irrigation management and dural closure, which require platform-specific adaptations and surgical expertise. ESS should be considered an emerging minimally invasive option rather than a replacement for established microsurgical approaches. However, these positive findings must be interpreted in the context of the small sample size, potential publication bias, and short follow-up length of 4–48 months, which may be inadequate to assess the recurrence of slower growing tumors. As such, prospective comparative studies are needed to better define its role in spinal oncology.

## Figures and Tables

**Table 1:** Patient Characteristics and Post-Operative Findings.

Study	Country	N	Age	F:M	Design	JBI	Symptoms	Length of Hospital Stay (Days)	Post-op Complication Rate (%)	Type of Complication	Reoperative Rate	Time to Adjuvant Therapy	Survival Rate at Time of Surgery (%)	Time to Follow-Up (Months)	Progression Free Survival (Months)
Zhang et al., 2023 [27]	China	5	61.2 ± 11.5	2:3	Case series	9/10	Pt 1- Lumbar pain + lower limb numbness, Pts 2,5- Thoracic pain + lower limb numbness/weakness, Pts 3,4- Lumbar pain	NR	20%	Temporary mild lower limb weakness	0%	NR	100%	25.2 ± 13.1	30
Jung et al., 2024 [28]	South Korea	1	78	1:0	Case report	8/8	Clumsiness, tingling in hands, gait disturbance, bilateral LE weakness	7 days	0%	N/A	0%	NR	100%	18	18
Liu et al., 2023 [29]	China	1	19	0:1	Case report	8/8	Sudden onset of low back pain, worse at night, relief with NSAIDs, no radiculopathy	<1 day	0%	N/A	0%	NR	100%	12	12
Hagel et al., 2024 [30]	Germany	1	46	1:0	Case report	8/8	Long-standing low back pain, bilateral LE pain, worsening of pain, bilateral feet weakness and hyperesthesia	2 days	0%	N/A	0%	NR	100%	16	16
Mangual-Perez et al., 2023 [31]	Puerto Rico	1	29	0:1	Case report	8/8	Thoracic pain, local tightness, pain at night, relief with NSAIDs	<1 day	0%	N/A	0%	NR	100%	12	12
Kravtsov et al., 2022 [32]	Russia	1	59	1:0	Case report	8/8	Constant leg pain, moderate paresis of left foot extensor, throbbing pain/paresthesia L5 dermatome, Lasègue sign	<1 day	0%	Paresis of the left extensor hallucis	0%	N/A (benign)	100%	48	48
Telfeian et al., 2015 [33]	USA	1	16	1:0	Case report	8/8	Mid thoracic back pain, left posterior rib pain, LE numbness	1 day	0%	N/A	0%	N/A (benign)	100%	NR	NR

JBI risk of bias tool is 10 items for case series and 8 items for case reports. The post-operative complications reported were not directly due to the procedure itself.

**Table 2:** Diagnostic and Surgical Information.

Variables (N = 11 Patients)	Number	Percentage
Primary spinal vertebral level		
Cervical	1	9.10%
Thoracic	4	36.36%
Lumbar	6	54.54%
Sacral	0	0%
Pathology		
Schwannoma	5	45.45%
Meningioma	3	27.27%
Osteoid osteoma	2	18.18%
Sarcoma	1	9.10%
Type of surgery		
Uniportal	5	45.45%
Biportal	1	9.10%
Tubular	5	45.5%
Tumor Resection		
Total resection	10	90.90%
Subtotal resection	0	0
Biopsy	1	9.10%
Primary tumor anatomical position		
Dorsal	3	27.26%
Ventral	1	9.10%
Lateral	2	18.18%
Dorsolateral	1	9.10%
Ventrolateral	3	27.26%
Foraminal	1	9.10%
Primary tumor dural location		
Intradural	7	63.64%
Extradural	4	36.36%

## Data Availability

Datasets are available upon request.

## Supplementary Materials

The following supporting information can be downloaded at https://www.mdpi.com/article/10.3390/jcm15124623/s1. PRISMA 2020 Checklist.

## Abbreviations

The following abbreviations are used in this manuscript:

MIS	minimally invasive surgery
ESS	endoscopic spine surgery
